# What Makes Young People Think Positively About Social Distancing During the Corona Crisis in Germany?

**DOI:** 10.3389/fsoc.2020.00061

**Published:** 2020-08-20

**Authors:** Marc Oliver Rieger

**Affiliations:** Department IV, University of Trier, Trier, Germany

**Keywords:** social distance, conspiracies, COVID 19, epidemic, prosocial behavior, SARS-CoV-2

## Abstract

In a survey among 250 subjects recruited at a German university and predominantly university students, we elicit opinions about social distancing, i. e., the necessity to keep away from other people to slow down the speed of the ongoing SARS-CoV-2 epidemics. The good news is that most students are supportive to it. A minority, however, does not completely agree. We find that how many elderly persons subjects knew personally, was the most significant factor for their attitudes toward social distancing. We also found a significant negative impact of believe in conspiracy theories on these attitudes. These theories have a non-negligible number of proponents, even among university students. Moreover, a certain degree of mistrust to media is widespread (around a third of the subjects). To improve positive attitudes to social distancing and thus to improve compliance we recommend therefore to emphasize relations of persons to elderly people in health communications more and to continue fighting against fake news and conspiracy theories regarding SARS-CoV-2.

## Introduction

The current pandemics of the new type of coronavirus, SARS-CoV-2, poses a global challenge. As of writing of this paper, in most countries around the world an exponential growth of cases and deaths is witnessed, with the notable exception of China where the disease started initially (David et al., 2020; Lu et al., 2020) and where it currently seems to be retained. In many countries, first in China, strict counter measures have been implemented, including closing of schools, universities and non-essential businesses as well as restricting individual mobility. In this way, the spread of the virus is supposed to slow down. Social distancing is key here: only if people don't meet with their friends and relatives, spreading can be reduced (Wilder-Smith and Freedman, 2020; Wu and McGoogan, 2020). And this can obviously only work if people comply with that.

Young people, and particularly university students, are particularly mobile and social. Their attitudes toward social distancing therefore plays an important role, especially in free and open societies where the ability to enforcement by state authorities is limited and traditionally obedience toward the state is less emphasized than in Confucian countries and in particular less free countries like China.

In order to measure the attitudes of university students in Germany toward social distancing, we conducted an online survey on March, 24 to March, 25 among 250 subjects, announced through the email information system of our university. While this sample is not representative, it is as close to a representative student sample as is possible given the needed urgency of this research.

We also elicited a number of potentially related factors (knowledge on COVID-19, beliefs in conspiracy theories around the disease, worries about the situation, and demographics), since some of these factors have previously been found to be interrelated:

There are a number of studies related to HIV demonstrating that more knowledge about the disease improves prevention measures among adolescents (DiClemente et al., 1986, 1988). Similar results have also been found in the context of influenza A/H1N1 (Lin et al., 2011), see also (Akan et al., 2010). In the case of COVID-19, the evidence is so far mixed: while (Imtiaz et al., 2020) finds a significant positive effect of education on precautionary behavior, they did not find an effect of better general knowledge about COVID-19. A better understanding of the nature of exponential growth, however, can motivate social distancing, as has been shown in controlled experiments (Lammers et al., 2020; Lunn et al., 2020). We therefore test not only general knowledge, but also particular knowledge about exponential growth in our study.

Conspiracy theories play an important and sometimes devastating effect on prevention measures against diseases. In particular, the case of HIV has been studied in details, see, e.g., Bogart and Thorburn (2005) and the book by Kalichman (2009). Another major concern are vaccination-related conspiracy theories (Jolley and Douglas, 2014), but the issue permeates all kind of health-related topics and influences various behaviors (Oliver and Wood, 2014). In the context of COVID-19, there are already first results on (psychological) factors increasing the receptiveness for conspiracy theories (Swami and Barron, 2020; Uscinski et al., 2020). Moreover, there are indications for a negative effect on the willingness to social distancing (Swami and Barron, 2020).

The problem of conspiracy theories becomes even more pressing as research has shown that it is much easier to prevent a belief in conspiracy theories to form than to undo it (Jolley and Douglas, 2017). We will study how widespread such beliefs are, in the case of COVID-19, and how they affect the willingness to social distancing and the worries about the situation.

The role of worries about COVID-19 has been emphasized in Pakpour and Griffiths (2020). Detailed measurements on factors increasing such worries have been elicited in the MERS outbreak (which was caused by a closely related virus and initially shared certain characteristics with the COVID-19 outbreak) in Ro et al. (2017), where it has been shown that particularly women and people with poor health conditions were worrying the most. We use a different set of items in our survey that allows to extend these results (in the context of the COVID-19 pandemic).

In the following, we describe the main questions of the survey (section Methodology), and present the key findings (section Main Results). Conclusions and practical advice are given in section Conclusions for Health Communication.

## Methodology

We recruited 250 subjects via the email information system of our university to participate in a short online survey that offered the chance to win 50€. The survey took 11 min to complete (median). It also contained a number of other questions not relevant for this paper.

Two hundred and four of our subjects were university students, the others mostly state employees, presumably at the university, although forwarding of the invitation to others was possible. Among the students 66% were female, the median age was 23 years.

In the following, we will only analyze results for the university students, where we removed 22 subjects, since they took a very short time (<5 min) to answer all questions, i.e., they might not have read the questions seriously, leaving a total sample of 182.

Eliciting actual behavior regarding social distancing in a standardized survey poses non-trivial challenges: one might expect a strong observer-expectancy or experimenter effect (Rosenthal, 1976) given that a lack of social distancing might increase the risk of spreading COVID-19 further and thus could be seen as an anti-social act. Following the theory of planned behavior (Ajzen, 1985), behavior (if caused by a conscious plan which is likely the case for most relevant situations) is caused by intention. While measuring intentions would cause similar problems as measuring behavior, its antecedents (attitudes, subjective norms, and perceived behavioral control), do not suffer as much from these problems. In our study, we focus on attitudes and subjective norms, as they can be measured in our survey avoiding a strong experimenter bias.

We therefore elicited attitudes (together with subjective norms) toward social distancing using a number of brief hypothetical “scenarios” about university students, taking place shortly before the general restrictions on private meetings in Germany were implemented^1^.

The scenarios were the following:

1. *A student celebrates a birthday party with his friends on the university campus. None of the friends belong to a risk group for coronavirus*.
2. *A student meets his friends to play soccer. None of them have cold symptoms*.
3. *Even though she has a cold, a student visits her grandmother in a nursing home, because her grandmother feels so alone*.
4. *A student refuses to hug her friends as usual when they meet to go jogging, and insists on keeping her distance, even though they tell her that they are not sick*.
5. *A student tells his friend that she finds it irresponsible for him to continue meeting his friends, even if he realizes that it hurts him*.

They were presented in randomized order. The students could judge each of them on a scale from 1 to 4, corresponding to “totally okay,” “not optimal, but understandable,” “rather bad,” “not acceptable.”

To capture their attitudes toward social distancing, the average of the items 4–5 was subtracted from the average of the items 1–3.

To measure the subject's knowledge on the new coronavirus, a number of statements were given that they should mark as correct or false, namely:

1. *Corona viruses can be spread by infected persons by coughing, sneezing, but also by speaking or breathing into the air*.
2. *Coronaviruses have been around for a long time, most of them cause harmless colds*.
3. *The correct name of the current pathogen is SARS-CoV-2*.
4. *The corona virus is a new form of flu virus*.
5. *Only those who suffer from symptoms can spread the virus further*.
6. *In most cases, the virus only leads to a mild illness*.
7. *The mortality rate of the infection is about 0.1% for young people, but in the double-digit percentage range for older people*.
8. *According to expert opinion, the development of a vaccine will take about 1 year*.

Here the statements 1, 2, 3, 6, 7, and 8 were correct, but 4 and 5 were wrong.

We computed a total score for each participant as sum of the correct choices.

We also asked a question about exponential growth:

If 1,000 people are infected in a country on 20 March, and 1000 more on 24 March, how many more infections can one expect a month later? (Assuming nothing is done to stop the spread.)

Here, exponential growth with a doubling of cases every 4 days (very similar to the rate for SARS-Cov-2 in Germany) leads to a correct answer of about 250,000. We considered an answer below 100,000 as underestimation of exponential growth and defined a dummy variable accordingly.

We measured the tendency to believe into conspiracy theories regarding COVID-19 with the following three items:

*I trust official information on the virus in Germany*.

*The media want to hide information about the virus from us*.

*The “hype” about Corona has only been caused by pharmaceutical companies to make money*.

In each case we asked for agreement on a four-point Likert scale (4 = agree fully, agree mostly, agree partially, 1 = disagree). A tendency to believe into conspiracy theories was defined as the sum of the scores for these items. While one *could* argue that a partial agreement to the first statement could be considered as a healthy dosage of doubt about politicians and authorities and not a tendency toward conspiracies, it seems difficult to say the same about the second and third statement. The total sum of these answers therefore seems to be a reasonable measurement instrument for our study.

We also elicited general worries about the corona virus situation, on a five-point Likert scale. Finally, we let subjects estimate the number of deaths that will occur in Germany until end of 2020 due to COVID-19 and the duration the current measures will have to be in place (in weeks).

## Main Results

### Social Distancing

**Figure 1:**
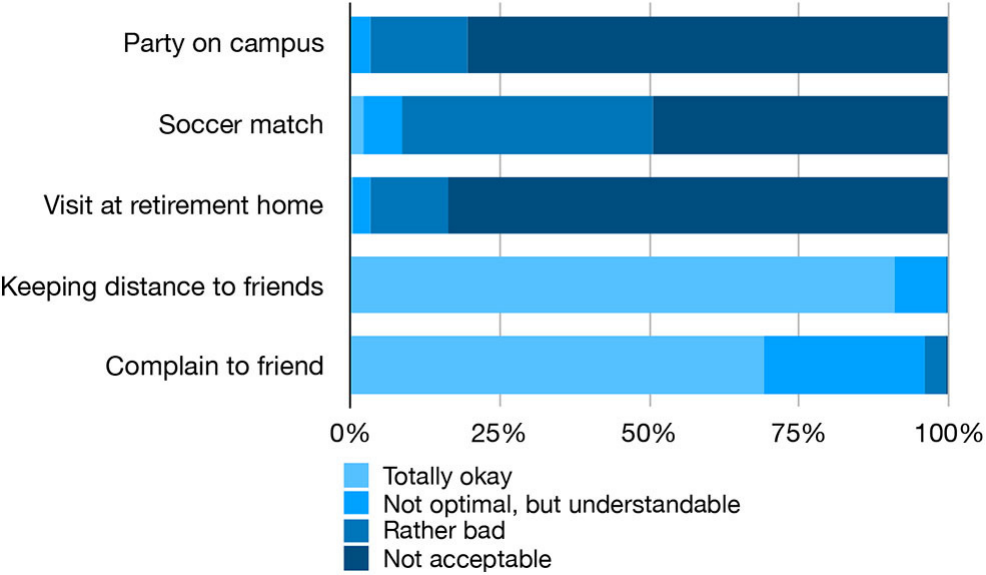
Evaluation of the four hypothetical scenarios as judged by the students. See main text for details.

The general attitudes of most subjects regarding social distancing are clearly supportive: in the three hypothetical scenarios, 80.3, 49.4, and 83.7% found the described behavior of people who violate social distancing “not acceptable,” while in the other two scenarios about students who insisted on social distancing, 91.0 and 69.1% found this behavior “totally okay.” There was, however, a small minority who disagreed entirely, in particular 8.9% thought that the soccer game was “total okay” or “not optimal, but understandable” and 4.0% thought it was “rather bad” or “not acceptable” to complain to a friend about him not conforming to social distancing (see Figure 1).

### Knowledge

The knowledge on the virus and the disease was good, but not excellent. Positive was that *all* subjects knew that the virus can also be transmitted by a person without symptoms. Quite a few subjects (41.4%) did not know that there are other, harmless coronaviruses. While this knowledge gap is not pivotal for health communication, the fact that 16.6% did not know that the virus can be spread “by coughing, sneezing, but also by speaking or breathing into the air,” is in this respect more worrisome.

Regarding the effects of exponential growth, the results are more negative: 39% strongly underestimated the number of cases in the example. Many subjects failed to see the effect of exponential growth. One out of eight students (12.9%) gave even values of 8,000 (a number that even a linear growth could predict) or lower. Only comparatively few subjects strongly overestimated the number.

### Conspiracy Theories

**Figure 2:**
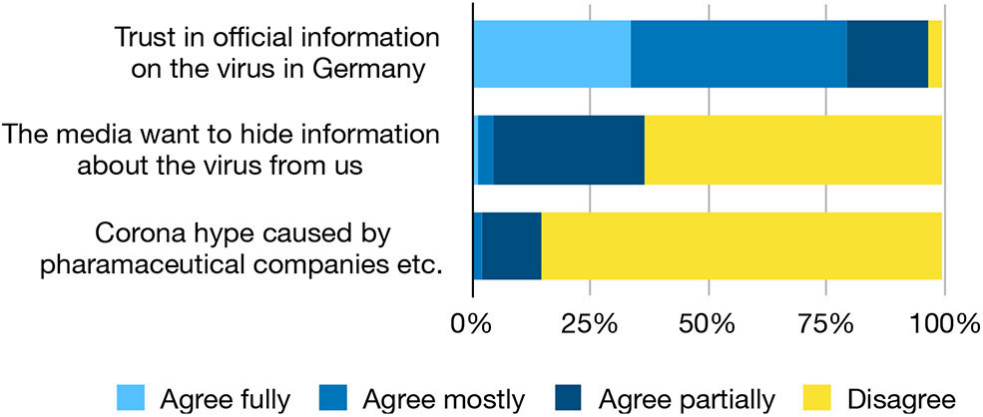
Trust in official information is fairly high, but there is a surprisingly large receptiveness for conspiracy theories.

A surprisingly large proportion of the subjects suspects that “the media” might intentionally retain information regarding the coronavirus: 36.5% agree to this at least partially (Figure 2). A small, but measurable number of students (14.6%) even agrees at least partially that everything is just a plot by pharmaceutical companies and “other interested groups.” This result is certainly worrisome.

### Estimates of Future Fatalities and Length

Subjects estimated the total number of deaths in Germany that will occur in 2020 due to COVID-19 as 3,000 (median). The estimates, however, varied wildly: from 100 to 2.4 million!

The estimated duration of the current restrictions was 6 weeks (median). Comparing both numbers to expert estimates that expect up to 70% of the German population to get infected over the course of the next 1–2 years (Robert-Koch-Institut, 2020) and given a case-fatality rate that has been estimated between 1.36 and 3.17% (Sun et al., 2020) suggests that the average student is rather optimistic in their estimates. We admit, however, that current estimates depend on many unforeseeable factors, e.g., the influence of seasonality on the virus, the availability of a vaccination, the age of the affected population and the potentially overestimation of the case-fatality rate due to undetected non-serious cases. However, already at the time of revising this article (June 4, 2020), 8,699 deaths have occurred, and many restrictions are still in place after more than 10 weeks.

### Reasons for Attitudes Toward Social Distancing

What influences attitudes toward social distancing? To this end, we conducted a regression analysis with several factors (the variables discussed previously) as dependent variable, see Table 1. After removing insignificant factors, we found that the following variables explain significantly the attitudes toward social distancing:

- The number of elderly people a subject personally knows,
- How much a subject believes in conspiracy theories.

**Table 1:** Influencing factors for attitudes toward social distancing.

	**Stand. beta**	***t*-value**	***p*-value**
Number of known old people	0.169	2.317	0.022
Acceptance of conspiracy theories	−0.208	−2.858	0.005
*R*^2^	6.4%		
*N*	180		

The second factor confirms recent results regarding the negative effect of conspiracy theories (Swami and Barron, 2020). The first factor can be explained by the availability bias (Tversky and Kahneman, 1973): if a person knows older people personally, he or she can more readily imagine the potentially deadly effect of COVID-19^2^. This may lead to an increased perceived severity of the disease and higher perceived benefits of its prevention. Drawing from the health belief model (see, e.g., Champion and Skinner, 2008), we would expect this to influence actual behavior, in this case on social distancing as preventive measure. Combining the health belief model with the aforementioned theory of planned behavior (Ajzen, 1985), the effect should not only influence actual social distancing behavior, but already the attitudes toward it. This could explain well why simply knowing more elderly people in person will help to improve attitudes toward social distancing. Utilizing this connection might be very helpful to improve compliance regarding social distancing.

Other factors did not play a significant role, neither personal worries, nor own estimations about deaths or duration. Knowledge played only a marginally significant role. This is in line with previous literature where a significant effect had been found in the case of HIV (DiClemente et al., 1986, 1988), but not in the case COVID-19 (Imtiaz et al., 2020). Contrary to Lunn et al. (2020) and Lammers et al. (2020), we did not find a significant effect of knowledge on exponential growth (this, however, could simply be a sample size effect).

### Why Young People Worry

Subjects worry about the coronavirus crisis: on a scale from 1 (very little) to 5 (very strongly), the average answer was 3.41 (±0.08), in other words, average students worry somewhere between medium and strongly.

A regression analysis (Table 2) shows that significant factors connected to these worries were:

- Estimated duration,
- The number of elderly people a subject personally knows,
- How much a subject believes in conspiracy theories.

**Table 2:** Factors influencing worries on the corona crisis.

	**Stand. beta**	***t*-value**	***p*-value**
Number of known old people	0.152	2.086	0.038
Estimated duration	0.242	3.353	0.001
Acceptance of conspiracy theories	−0.215	−2.903	0.004
Female	0.137	1.849	0.066
*R*^2^	13.4%		
*N*	170		

The first two factors enhance previous related results on MERS (Ro et al., 2017) and fit into the overall picture that worries increase when a subject will more likely be personally affected (either directly due to health conditions as in Ro et al. (2017) or indirectly due to knowing people who might be more affected by the disease due to their age or simply by a longer duration of a lock-down.

Moreover, women tend to state higher degrees of worries, as in a previous study on MERS (Ro et al., 2017), but this effect was not significant at 5%. Conspiracy theories actually reduced the worries, it seems because subjects that believed in them found the whole crisis to be overexaggerated and therefore not much of a concern (Rieger and Wang, 2020).

Interestingly, knowing a case personally, did not change the amount of worries significantly, neither did knowledge on the virus.

## Conclusions for Health Communication

In this article we have presented results from a current survey on attitudes of German university students regarding social distancing in the context of the ongoing coronavirus crisis. We have focused on empirical results and applications, but more theoretical work on macro- and fundamental issues would be possible as well, but is beyond the scope of this study.

We have seen that the majority of participants strongly supports social distancing measures, but there is a non-negligible number that sees things less clear cut, and some believe in outright conspiracy theories about COVID-19. This, together with some gaps in their knowledge on SARS-CoV-2 and on the expected effects of exponential growth on the further curse of the epidemics is definitely problematic and needs to be addressed in health communication.

Factors that make students have a more positive attitude about social distancing are in particular the number of elderly people they know (as they are known to be at highest risk). Belief in conspiracy theories, however, causes the opposite. This suggests two ways for health communication to increase compliance with social distancing:

- Letting young people connect better to the worries of people at risk, particularly the elderly,
- Actively combating conspiracy theories.

(in a follow-up study, we are going to take a closer look at the origins of conspiracy theories in the context of COVID-19 and their effect on precautionary behavior).

Another practical consequence from our study is that we should not worry about confronting people who violate social distancing: even among young people like university students most support such confronting behavior.

## Data Availability Statement

The raw data supporting the conclusions of this article will be made available by the author, without undue reservation.

## Ethics Statement

Ethical review and approval was not required for the study on human participants in accordance with the local legislation and institutional requirements. Written informed consent for participation was not required for this study in accordance with the national legislation and the institutional requirements.

## Author Contributions

The author confirms being the sole contributor of this work and has approved it for publication.

## Conflict of Interest

The author declares that the research was conducted in the absence of any commercial or financial relationships that could be construed as a potential conflict of interest.

## Footnotes

^1^Scenarios were set to have taken place on the weekend of March 21 and 22; the rules were implemented on March 23 and 24. The survey was conducted on March 24 and 25.

^2^Similar effects of the availability bias have been found in other health related situations, e.g., differences in perceived urgency of symptoms between patients and doctors can be attributed to this availability bias (Peay and Peay, 1998), and the bias also leads to an increase of personal breast cancer risk estimates when a close person had been sick (Chalmers and Thomson, 1996).
